# Dose-related cardiac side effects and its impact on survival following thoracic radiotherapy: A scoping review

**DOI:** 10.1016/j.ctro.2026.101304

**Published:** 2026-10-06

**Authors:** Umut Fidan, Arno C. Hessels, Anne P.G. Crijns, Robin Wijsman, Peter van der Meer, Johannes A. Langendijk, Christina T. Muijs, Peter van Luijk

**Affiliations:** a Department of Radiation Oncology, University Medical Centre Groningen, Hanzeplein 1, P.O. Box 30001, 9700, RB, Groningen, the Netherlands; b Department of Cardiology, University Medical Centre Groningen, Hanzeplein 1, P.O. Box 30001, 9700, RB, Groningen, the Netherlands

**Keywords:** Radiation-induced cardiovascular toxicity, Thoracic cancer, Dose-response relationship

## Abstract

Radiation dose to the heart is associated with various cardiac side effects. However, the specific cardiac and pulmonary structures that contribute to these toxicities remain incompletely understood. Moreover, although multiple factors contribute to reduced survival after radiotherapy, the role of radiation-induced cardiac toxicity remains underrecognized. This scoping review aimed to evaluate the associations between radiation dose to specific cardiac and pulmonary structures, cardiac side effects, and survival.

A total of 137 clinical studies were analysed in accordance with the PRISMA guidelines. Associations between radiation dose and cardiac side effects were identified not only for the whole heart but also for specific cardiac substructures, including myocardial segments, coronary arteries, and the pericardium. Notably, radiation dose to the lungs was also associated with decline in cardiac function, suggesting that minimizing cardiac toxicity may require consideration of both local cardiac irradiation and non-local irradiation of adjacent organs, particularly the lungs. Direct evidence linking cardiac side effects to survival was limited. Nevertheless, the observed associations between higher radiation doses, increased cardiac toxicity, and poorer survival suggest a potential relationship that warrants further investigation.

These findings support the hypothesis that reducing specific cardiac side effects may benefit from targeted sparing of the relevant cardiac and pulmonary substructures. While many radiation-related cardiac side effects are now recognized, further research is needed to identify and characterize previously unrecognized manifestations and to clarify their contribution to long-term survival.

## Highlights

• Thoracic radiotherapy can cause a broad spectrum of cardiac side-effects.
• These are associated with dose to specific cardiac substructures and/or the lungs.
• Targeted sparing of these may reduce cardiac side effects and may improve survival.

## 1 Introduction

Radiotherapy plays an important role in the curative treatment of (locally advanced) thoracic malignancies [1], [2], [3], [4]. Radiotherapy improves survival and tumour control by inducing direct and indirect DNA damage, leading to cancer cell death [5], [6]. However, inevitable radiation exposure of heart and lungs may lead to cardiac side effects [7], [8]. These include myocardial fibrosis, reduced cardiac systolic function, coronary artery diseases, pericarditis, arrhythmia, and valvular disorders [7], [8], [9], [10], [11]. These side effects can significantly impact quality of life, and even survival, thereby partly compromising the benefits of radiotherapy [12], [13].

The risk of cardiac toxicity is dose-dependent, with radiation dose to the heart or its substructures playing a role in its development [14], [15], [16]. Moreover, studies in thoracic cancer patients have shown associations between radiation dose to heart and/or lungs and survival [17], [18], [19]. Although several factors contribute to mortality following thoracic radiotherapy, including pre-existing cardiac comorbidities, pulmonary toxicity, and radiation-induced lymphopenia [20], [21], [22], cardiac toxicity remain a critical and potentially underrecognized mechanism linking radiation dose with survival outcomes.

Despite these observations, treatment-related risk factors, such as radiation dose and irradiated volume of organs at risk (OARs), have not yet been clearly established for individual radiation-induced cardiac side effects. Furthermore, the relationship between radiotherapy-associated cardiac toxicity and survival outcomes remains poorly understood.

Therefore, this review aims to inventory the cardiac side effects of thoracic radiotherapy, assess their relationship with radiation dose to cardiac/pulmonary structures and assess their impact on survival.

## 2 Methods

This scoping review followed the PRISMA-ScR (Preferred Reporting Items for Systematic Reviews and Meta-Analyses extension for Scoping Reviews) checklist [23]. A structured, comprehensive search was carried out in the PubMed/MEDLINE, Cochrane, EMBASE and Web of Science Core Collection databases for all articles published until February 4, 2026.

Studies were included if they: (1) involved adult patients with thoracic cancers treated with radiotherapy; (2) analysed radiation dose to organs at risk; (3) reported cardiac side effects separately; and (4) were published in English. Exclusion criteria included stereotactic ablative radiotherapy, case studies, editorials, meeting abstracts, and reviews. Stereotactic ablative radiotherapy was excluded because of its very high doses per fraction over a shorter treatment course, expected to result in dose-effect relationships that differ significantly from those occurring in conventional radiotherapy [24].

The search strategy included terms related to thoracic malignities, radiotherapy dose, and cardiac side effects and is available in the Supplementary Table 1. Title/abstract screening, full-text eligibility assessment, and data extraction were performed by a single reviewer (UF), using Rayyan [25]. Fig. 1 summarizes the study selection process, including duplicate removal and reasons for full-text exclusion. Extracted data included study characteristics (e.g., publication year, patient population, follow-up length, and study endpoints), cardiac dose parameters, and their associations with cardiac outcomes and survival. Supplementary Table 2 summarizes the characteristics and main findings of the included studies, including reported effect estimates and 95% confidence intervals where available. In accordance with the objectives of this scoping review and PRISMA-ScR, no formal risk-of-bias assessment of individual studies was performed.

**Fig. 1:**
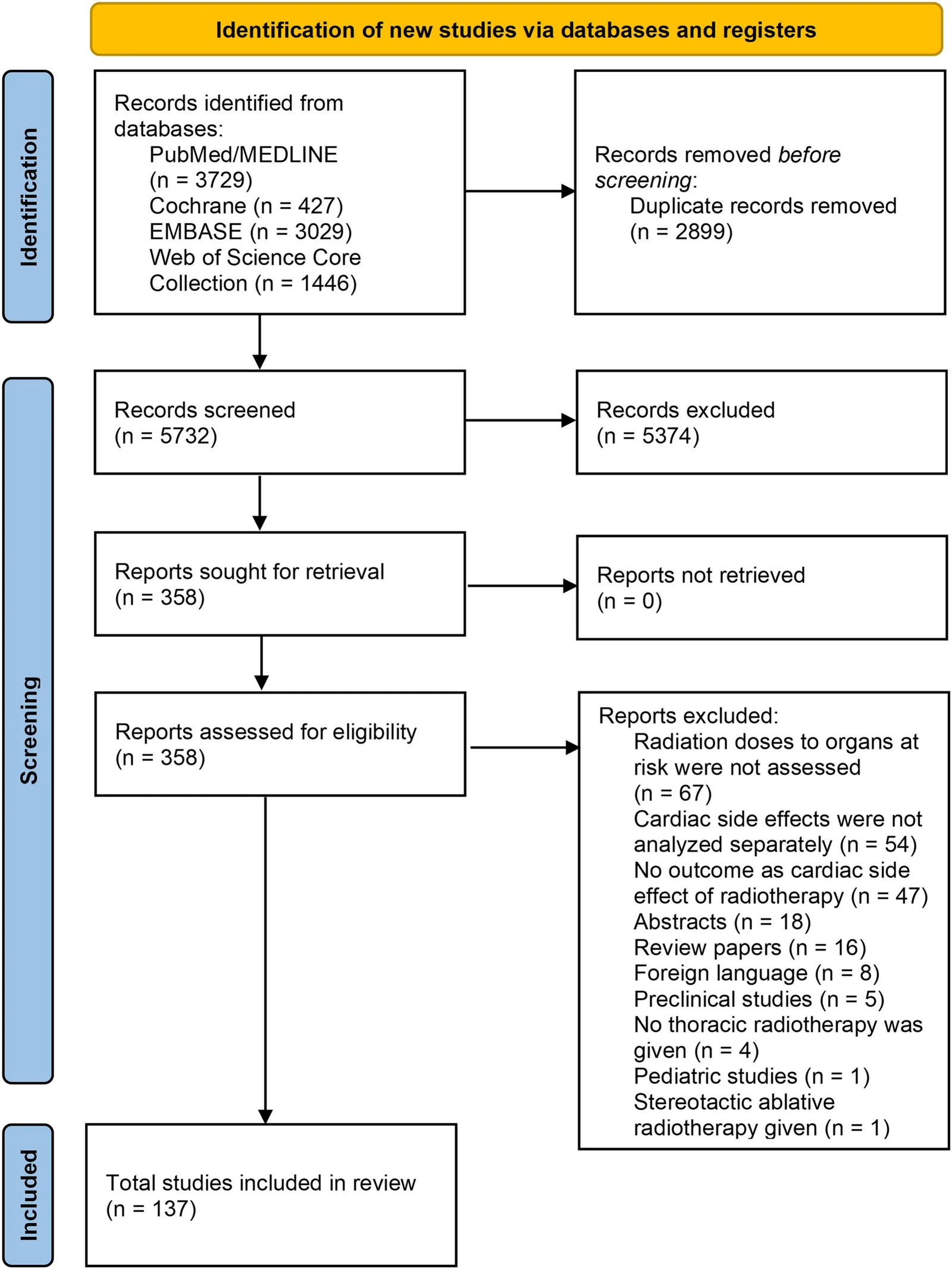
PRISMA flowchart of the article selection process.

## 3 Results - Dose to normal tissues and potential relation to OS?

### 3.1 Cardiac side effects

This scoping review aimed to identify cardiac side effects associated with thoracic radiotherapy, assess their relationship with radiation dose and evaluate their impact on OS. Due to the diversity of cardiac side effects, dose-effect relationships were analysed separately for each outcome and subsequently summarized. Additionally, dose-response relationships and their potential impact on OS were reviewed.

A total of 5732 articles were screened based on title and abstract, of which 5595 were excluded for not meeting the inclusion criteria. Ultimately, 137 articles were included in the review (Fig. 1).

#### 3.1.1 Myocardial damage

Clinical and preclinical studies suggest that myocardial damage can develop within weeks after radiotherapy and may progress over time [[9], [10], [26], [27]]. Most clinical studies evaluating dose-effect relationships reported a significant association between myocardial damage and radiation dose to the heart [[28], [29], [30], [31], [32], [33], [34], [35], [36], [37], [38], [39], [40], [41], [42], [43], [44]] (Supplementary Table 2). In addition to whole-heart dose, several studies reported associations with radiation dose to specific cardiac substructures, particularly the left ventricle [[35], [45], [46], [47]], and coronary arteries [[48], [49], [50], [51]].

Not all studies observed a significant dose-effect relationship. This may be attributable to limited statistical power resulting from small cohort sizes [[52], [53], [54]] or to the relatively low cardiac doses in breast cancer cohorts, where the average mean heart dose (MHD) was approximately 2.0 Gy [55]. Several studies also suggested dose thresholds for myocardial changes [[41], [42], [46], [56], [57], [58]]. However, these thresholds varied considerably across studies, likely reflecting differences in the assessed endpoints, which ranged from metabolic changes to myocardial injury.

Preclinical evidence suggest that radiation exposure to the lungs may also contribute to ventricular remodelling and dysfunction [59]. In line with this, two studies identified associations between MLD and impaired posterior wall contractibility as well as myocardial fibrosis [[35], [36]]. In addition, one study identified an association between MLD and elevated NT-proBNP levels [38], a biomarker of cardiac stress. Although these observations may suggest that lung irradiation plays a role in the development of cardiac damage, the current evidence remains limited. Larger, well powered studies are required to establish whether the lung should be considered an organ at risk for the development of myocardial damage.

Although the mechanisms underlying myocardial damage are not yet fully understood, local radiation dose has been associated with fibrotic remodelling [[40], [60]] as well as changes in myocardial metabolism and perfusion [[56], [57], [58], [61], [62], [63]]. Perfusion abnormalities may, in part, result from radiation induced endothelial injury and inflammatory responses in the coronary vasculature, consistent with the reported associations between myocardial damage and dose to the coronary arteries [[48], [49], [50], [51]].

Taken together, the available evidence suggests that myocardial damage is associated with radiation doses delivered to the heart, especially specific myocardial substructures. Such localized dose–effect relationships have not been consistently observed in breast cancer cohorts, possibly because breast radiotherapy typically results in lower mean heart doses and smaller irradiated cardiac volumes.

#### 3.1.2 Valvular heart disease

Valvular heart disease is common after radiotherapy, with e.g., a prevalence of 30-50% among lymphoma patients within 10-20 year after treatment [[64], [65], [66], [67], [68]]. Association with radiation dose to the heart, or more specifically the valves (Supplementary Table 2), suggest that radiotherapy plays a role in this [[64], [65], [66], [67], [68], [69]]. Only one study did not find a relationship with dose, likely due to a low number of valvular disease events observed (*n* = 3) [70]. Most of these studies were performed in lymphoma patients, likely due to the long latency of valvular disease exceeding the typical life expectancy of patients with lung or oesophageal cancer [[71], [72], [73]].

Notably, two studies also identified a potential link between lung irradiation and valvular disease [[65], [66]]. However, as the underlying mechanism remains unclear and lung dose may be correlated with cardiac dose and other treatment-related factors, these observations should be interpreted cautiously.

#### 3.1.3 Coronary artery disease

Multiple studies observed associations of coronary disease with heart dose with no apparent dose threshold (Supplementary Table 2) [[69], [74], [75], [76], [77], [78], [79], [80], [81], [82], [83]]. Also, coronary disease was associated with radiation dose to the left ventricle [[84], [85], [86], [87]]. Since strong correlations exist between cardiac doses and dose to coronary arteries, particularly to the left anterior descending coronary artery (LAD) and right coronary artery (RCA) [[74], [88]], both associations with heart dose and left ventricle dose likely reflect the effect of coronary artery dose, as indicated by observed associations with radiation doses to the LAD [[79], [89], [90], [91], [92], [93], [94]], as well as RCA, left main coronary artery (LMCA) and left circumflex (LCX) artery [[89], [91], [92], [94]]. In line with this, radiation doses to specific coronary regions were observed to be associated with region-specific atherosclerotic changes [[85], [95]]. Overall, these data suggest that coronary artery damage is related to radiation dose to the coronary arteries. If a threshold dose exists, it is low and not yet accurately identifiable from the available data.

Interestingly, one study observed an association between dose in pre-existing plaques in the coronary arteries and coronary events [85]. Although it is not confirmed [78], specifically reducing dose to plaques might reduce the risk of coronary events [85].

However, in two other studies, the risk of coronary events did not increase with higher radiation doses to the RCA or LCX [[90], [96]], which may be due to lower overall cardiac doses in that study (median MHD of <1.5 Gy).

#### 3.1.4 Pericardial complications

With an incidence exceeding 20%, pericardial disease is the most common manifestation of radiation-induced heart disease [8], [9], [10]. Pericardial effusion is the most frequently observed pericardial disease after radiotherapy [[86], [97], [98], [99], [100], [101], [102], [103], [104], [105], [106], [107], [108], [109], [110], [111], [112], [113]]. Most studies evaluating dose–effect relationships identified significant associations between pericardial disease and radiation dose to the heart [[70], [86], [97], [98], [100], [101], [103], [104], [105], [108], [109], [111], [112], [113]] as well as the pericardium [[98], [100], [101], [102], [105], [106], [107], [110], [112]] (Supplementary Table 2). Only one study, which included a limited number of events (*n* = 5), found no association with radiation dose [114]. Although heart and pericardial dose metrics are strongly correlated [[100], [102], [112]], studies that evaluated both generally found stronger associations for pericardial dose. These findings suggest that the pericardium, rather than the heart as a whole, is the primary organ at risk for the development of radiation-induced pericardial disease [[98], [100], [101], [102], [105], [106], [107], [110], [112]].

#### 3.1.5 Arrhythmia

Arrhythmias are common in the general population [114]. Nevertheless, several studies have reported associations between arrhythmia risk and radiation dose to the heart [[86], [104], [115]]. As different types of arrhythmias originate from distinct anatomical regions of the cardiac conduction system, radiation-induced arrhythmias are likely driven by damage to specific cardiac substructures rather than by whole-heart exposure alone. Consistent with this hypothesis, multiple studies have identified associations between individual arrhythmia types and radiation dose to cardiac substructures involved in their pathogenesis or located in proximity to known arrhythmogenic regions (Fig. 2) [[14], [70], [86], [99], [104], [115], [116], [117], [118], [119], [120]].

**Fig. 2:**
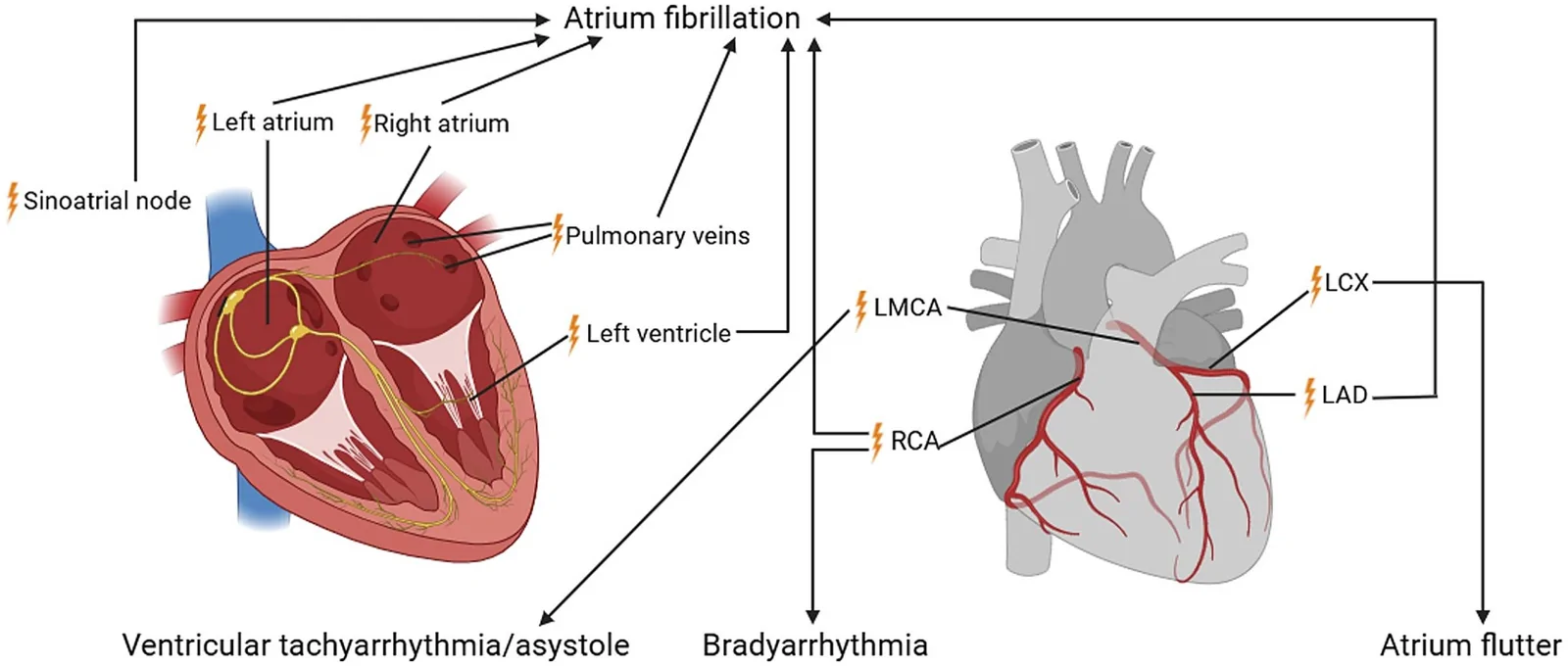
Forms of arrhythmia with associated structures at risk. LAD = left anterior descending coronary artery, LCX = left circumflex coronary artery, LMCA = left main coronary artery, RCA = right coronary artery.

Not all studies observed significant dose-effect relationships. These negative findings may be explained by low event rates, limited statistical power, or relative low radiation doses to critical cardiac structures [[96], [114], [121], [122]]. In addition, non-radiation treatment factors may contribute to arrhythmia risk. Surgical is an important confounder and was associated with an approximately twofold risk of atrial fibrillation among oesophageal cancer patients, in addition to cardiac radiation dose parameters, in the study by Miller et al. [115].

#### 3.1.6 Ventricular dysfunction

Ventricular dysfunction is thought to result from radiation-induced damage to one or more cardiac substructures involved in myocardial contractility. In line with this hypothesis, multiple studies have reported associations between post-radiotherapy ventricular dysfunction and dose to the heart (Supplementary Table 2) [[37], [41], [48], [69], [122], [123], [124], [125], [126], [127], [128], [129], [130], [131], [132]]. Associations with radiation dose to the ventricles further support the notion that ventricular dysfunction is predominantly driven by localized myocardial injury, rather than by diffuse effects of whole-heart irradiation [[52], [93], [96], [122], [124], [126], [128], [130], [131], [133], [134], [135], [136], [137], [138], [139], [140], [141], [142], [143], [144], [145]]. This is further supported by four studies showing that regional loss of ventricular function correlates with the radiation dose delivered to the affected ventricular segments [[146], [147], [148], [149]], suggesting a clear spatial relationship between local radiation exposure and functional impairment.

Furthermore, multiple studies reported associations between ventricular dysfunction and radiation dose to the coronary arteries, most notably the LAD [[96], [133], [135], [143], [144], [150], [151], [152]]. This suggests that impaired myocardial perfusion secondary to radiation-induced coronary artery damage may contribute to the development of ventricular dysfunction [152]. However, interpretation of these associations is complicated by the anatomical proximity of coronary arteries and ventricles, which usually results in correlated radiation doses to both structures.

On the other hand, several studies did not report associations with radiation dose. This is likely explained by low event rates either due to small sample size [[55], [153], [154], [155], [156], [157], [158]], or lower doses to critical structures [[55], [153], [154], [155], [156], [157], [159], [160], [161], [162]]. Indeed, studies reporting no significant associations generally involved low cardiac exposures, with average MHDs below 4 Gy (9 out of 11 studies).

#### 3.1.7 Beyond known side effects

In addition to the cardiac toxicities discussed above, several potential radiation-induced cardiac effects remain insufficiently characterized. For example, three studies reported cardiac volume reductions during or shortly after radiotherapy in oesophageal cancer patients. However, no associations with cardiac dose were observed potentially due to small sample size [[163], [164], [165]]. In addition, one large study (*n* = 848) identified an association between pulmonary artery dose and pulmonary hypertension [166]. Both effects can potentially impact patient health [[167], [168]].

Improved understanding of the underlying mechanisms, the specific structures at risk, and the corresponding dose constraints may therefore contribute to better prevention of these complications and improved clinical outcomes. More broadly, these findings highlight that the full spectrum of radiation-induced cardiac toxicity may not yet be completely understood, and that additional, currently underrecognized cardiac side effects may emerge as evidence continues to accumulate.

### 3.2 Survival

As evident from the reviewed literature, thoracic radiotherapy can induce a wide range of cardiac side effects. An important question is, however, whether these side-effects translate into worse patient prognosis. To address this question, we distinguish between direct evidence evaluating associations between cardiac toxicity and survival, and indirect evidence evaluating associations between radiation dose and survival.

Several studies directly evaluated associations between cardiac endpoints and survival. Prospective studies reported associations between myocardial biomarkers, including NT-proBNP and Troponin-T, and OS [44] as well as cardiac-related deaths [38]. Similarly, impaired right ventricle function has been associated with poorer OS [131]. One study suggested that a higher radiation dose to the right atrium was associated with atrial fibrillation, which in turn may be associated with poorer OS [115].

Interestingly, one study reported that the onset of AF following radiotherapy was associated with improved survival, even after adjustment for clinically relevant comorbidities [119]. The authors hypothesized that this unexpected finding may be explained by increased clinical surveillance and more intensive management of cardiovascular risk factors following AF diagnosis [119].

In contrast, several studies found no significant associations between radiation-induced cardiac side effects and survival [[39], [61], [98], [112], [115], [120]]. Interpretation of these findings is limited by, among other factors, small patient numbers [39] and low event rates [120]. Furthermore, particularly in patients with thoracic malignancies, OS is determined by multiple competing prognostic factors, including age, tumour stage, treatment response, and pre-existing comorbidities [[82], [91]]. Consequently, establishing an independent contribution of cardiac toxicity to OS remains challenging.

Separate from studies directly evaluating cardiac toxicity and survival, several studies investigated associations between radiation dose to thoracic structures and survival outcomes. Associations between dose metrics to the heart, lungs, pericardium, and cardiac substructures, including the left atrium, and poorer OS have been reported [[14], [76], [91], [98], [112], [115], [116]]. Associations between radiation dose and cardiac-related mortality have also been described [[74], [88]].

Although these findings suggest a relationship between thoracic radiation exposure and survival, they do not establish that radiation-induced cardiac toxicity mediates this association.

This distinction is particularly important in lung and oesophageal cancer, where survival is influenced by numerous disease-, patient-, and treatment-related factors. Cardiac and pulmonary dose may be associated with tumour burden, target volume, pulmonary toxicity, lymphopenia, and comorbidity, making it difficult to distinguish a potential effect of radiation-induced cardiopulmonary injury from other causes of poorer survival. Moreover, cardiac and pulmonary dose parameters are often correlated with each other and with treatment characteristics. Residual confounding and multicollinearity may therefore contribute to the observed associations between thoracic dose parameters and survival. Consequently, dose–survival associations should be considered indirect evidence and interpreted cautiously with regard to the contribution of radiation-induced cardiac toxicity.

Overall, the current evidence remains inconclusive regarding the extent to which specific radiation-induced cardiac side effects affect survival. While several studies suggest that cardiac injury may contribute to reduced OS and increased cardiac mortality, disentangling these effects from other prognostic factors remains challenging. Future large-scale, prospective studies assessing individual cardiac endpoints enabling multivariable survival analyses are needed. This may clarify the independent impact of cardiac side effects on survival and whether preventing specific side effects may improve long-term outcomes.

## 4 Discussion

This scoping review evaluated the relation between radiation dose to organs-at-risk and the development of cardiac side effects following thoracic radiotherapy, as well as their impact on OS. The available evidence consistently demonstrates that thoracic irradiation is associated with a broad spectrum of cardiac complications (Table 1). Importantly, most of these toxicities show clear dose–response relationships, underscoring cardiac injury as a clinically relevant and dose-dependent consequence of thoracic irradiation.

**Table 1:** Key findings across cardiac side effects.

Cardiac side effect	Size of study population	Studied tumour types	Follow-up length	Significant associations reported with cardiac dose (Y/N)	Associated cardiac substructure dose	Significant associations reported with lung dose (Y/N)	Type of analysis
Myocardial damage [[28], [29], [30], [31], [32], [33], [34], [35], [36], [37], [38], [39], [40], [41], [42], [43], [44], [45], [46], [47], [48], [49], [50], [51], [52], [53], [54], [55], [56], [57], [58], [60], [61], [62], [63], [169]]	12-482	Breast (*n* = 15), Esophagus (*n* = 13), Lung (*n* = 4), Mixed (n = 4)	From start of radiotherapy to 87 months	Yes (*n* = 19/36)	- Coronary arteries, such as LAD and LCX (n = 4) - Chambers of heart (LA, LV, RA, RV) (*n* = 8) - Myocardial segments (n = 8)	Yes (*n* = 3)	Univariable: 29/36 Multivariable: 7/36
Valvular heart disease [[64], [65], [66], [67], [68], [69], [70]]	63-14,645	Breast (*n* = 1), Lymphoma (*n* = 5), Lung (n = 1)	70-225 months	Yes (n = 5/7)	- Chambers of heart (LA, RV) (n = 1) - Affected valve (n = 1)	Yes (*n* = 2)	Univariable: 5/7 Multivariable: 2/7
Coronary artery disease [[69], [74], [75], [76], [77], [78], [79], [80], [81], [82], [83], [84], [85], [86], [87], [88], [89], [90], [91], [92], [93], [94], [95], [96]]	20-14,645	Breast (*n* = 14), Esophagus (n = 1), Lymphoma (*n* = 3), Lung (*n* = 4), Mixed (*n* = 2)	From 12 months to above 20 years	Yes (n = 14/24)	- Chambers of heart (LA, LV, RV) (*n* = 7) - Aorta and pulmonary artery (n = 1) - Coronary arteries (n = 7) - Segments of coronary arteries (n = 3)	Not shown	Univariable: 4/24 Multivariable: 20/24
Pericardial complications [[70], [86], [97], [98], [99], [100], [101], [102], [103], [104], [105], [106], [107], [108], [109], [110], [111], [112], [113], [114]]	69-350	Breast (n = 1), Esophagus (*n* = 13), Lung (*n* = 6)	8-105 months	Yes (*n* = 14/20)	- Chambers of heart (LA, RA, RV) (*n* = 3) - Coronary arteries (such as LAD) (*n* = 2) - Pericardium (*n* = 9)	Not shown	Univariable: 7/20 Multivariable: 13/20
[[14], [70], [86], [96], [99], [104], [114], [115], [116], [117], [118], [119], [120], [121], [122]]	112-748	Breast (n = 2), Esophagus (*n* = 4), Lung (*n* = 8), Mixed (*n* = 1)	12-105 months	Yes (*n* = 3/15)	- Chambers of heart (LA, RA) (n = 4) - Coronary arteries (LAD, LCX, LMCA, RCA) (n = 3) - Sinoatrial node (n = 1) - (Left and right) pulmonary vein (n = 3)	Not shown	Univariable: 4/15 Multivariable: 11/15
Ventricular dysfunction [[37], [41], [48], [52], [55], [69], [93], [96], [122], [123], [124], [125], [126], [127], [128], [129], [130], [131], [132], [133], [134], [135], [136], [137], [138], [139], [140], [141], [142], [143], [144], [145], [146], [147], [148], [149], [150], [151], [152], [153], [154], [155], [156], [157], [158], [159], [160], [161], [162]]	10-14,645	Breast (*n* = 35), Esophagus (*n* = 4), Lung (*n* = 5), Lymphoma (*n* = 1), Mixed (n = 4)	From start of radiotherapy to 168 months	Yes (*n* = 21/49)	- Chambers of heart (LV, RV) (*n* = 19) - Coronary arteries (LAD, LCX, LMCA) (*n* = 9) - Left ventricular segments (n = 4)	Not shown	Univariable: 30/49 Multivariable: 19/49

LA = left atrium, LV = left ventricle, RA = right atrium, RV = right ventricle, LAD = left anterior descending coronary artery, LCX = left circumflex coronary artery, LMCA = left main coronary artery, RCA = right coronary artery.

An interesting observation from this review is that many radiation-induced cardiac toxicities appear to be more closely associated with dose to specific cardiac substructures than with whole-heart dose metrics. For example, myocardial damage and ventricular dysfunction are predominantly linked to radiation exposure of ventricular segments, while coronary artery disease correlates closely with dose to individual coronary arteries, particularly the left anterior descending artery (LAD). Similarly, arrhythmias are associated with irradiation of atrial regions and conduction-related structures, and pericardial complications with pericardial dose. These findings suggest that global heart dose metrics may obscure clinically relevant local effects, and that substructure-based dosimetry provides a more precise understanding of radiation-induced cardiac injury.

This also has important implications for treatment planning. Although reducing MHD generally lowers dose exposure to all cardiac substructures, certain regions may have a greater impact on toxicity and should therefore be prioritized. Planning studies suggest that targeted sparing of cardiac substructures can achieve greater dose reductions in critical regions than whole-heart–based approaches [170]. The potential clinical relevance of such approaches is supported by studies showing that radiation dose to specific cardiac substructures, such as the heart base in lung cancer and the left anterior descending artery (LAD) in breast cancer, correlates more strongly with overall survival (OS) than mean heart dose (MHD) [[88], [171]]. Building on this concept, the Manchester group adopted a complementary strategy by identifying cardiac substructures whose radiation exposure was associated with OS, rather than focusing on individual cardiac toxicities [171], [172], [173]. This approach provides valuable insight into which cardiac regions may be particularly important determinants of long-term survival and therefore warrant enhanced protection during treatment planning.

In addition to cardiac dose, this review suggests that lung irradiation may warrant consideration when evaluating cardiac toxicity. Preclinical studies have suggested that lung irradiation could contribute to non-local cardiac damage, potentially through inflammatory and microvascular mechanisms [[59], [174], [175]]. Clinically, several studies have reported associations between lung dose and cardiac outcomes, including myocardial damage and valvular disease [[35], [36], [38], [65]]. However, these findings should be interpreted with caution, as lung and cardiac dose parameters are often correlated. Multicollinearity may therefore partly explain observed associations with lung dose and makes it difficult to establish whether lung irradiation contributes independently to cardiac toxicity. Further studies are needed to disentangle the respective contributions of cardiac and pulmonary irradiation and to determine whether incorporating lung dose parameters provides additional value in prediction models.

In addition to dosimetric factors, patient and treatment characteristics may substantially influence the risk of cardiac toxicity and subsequent survival outcomes [176]. As noted previously, surgical resection is an important confounder of the association between radiation exposure and AF in patients with oesophageal cancer [115]. Furthermore, several clinical studies have consistently identified cardiovascular comorbidities as an important confounder of cardiac complications and reduced OS [[82], [172], [177]]. However, adjustment for these and other relevant factors varied considerably across studies, and many analyses relied primarily on univariable methods. Inadequate adjustment for potential confounders may therefore partly account for some of the reported dose-response associations.

Beyond their potential confounding effects, clinical risk factors may also modify the relationship between radiation exposure and cardiac outcomes. However, such interactions remain poorly understood and have not been systematically investigated. Further research is needed to clarify whether and how they modify dose–response relationships. Improved understanding of these interactions could facilitate more personalized risk stratification and treatment planning.

In addition, although knowledge of radiation induced cardiac side effects has markedly increased over the past decade, we may still not fully recognize all effects. For instance, irradiated rats developed morphological and vascular changes in lungs, leading to pulmonary hypertension [[59], [174], [175], [178]]. Clinically, dose-dependent changes in pulmonary arterial pressure [[166], [179]] were observed. Moreover pulmonary hypertension was associated with longer hospital stay and mortality in patients who were admitted to the hospital for radiation pneumonitis [180]. In rats, combined irradiation of the heart and lungs produced a greater impairment of cardiac function and more severe pulmonary hypertension than irradiation of either the heart or lungs alone [[59], [178]]. Together these findings underscore the need for a comprehensive treatment plan optimisation approach that considers the risk of multiple side effects and their interactions, as well their dependence on non-radiotherapy factors such as comorbidities.

Advances in radiotherapy techniques have substantially increased treatment precision and reduced radiation exposure to normal tissues. Compared with Three-Dimensional Conformal Radiation Therapy (3D-CRT), more advanced techniques such as Intensity-Modulated Radiation Therapy (IMRT) and Volumetric Modulated Arc Therapy (VMAT) enable improved dose conformality around the target while reducing high dose exposure to surrounding healthy tissues, translating into lower toxicity and improved survival [[181], [182]]. Due to its distinct beam properties, proton radiotherapy further facilitates dose reductions to heart and lungs [[182], [183], [184]]. In oesophageal cancer, a randomized phase IIb trial reported a sevenfold reduction in postoperative complications with proton therapy compared with IMRT [185], while meta-analytic data suggest reductions in cardiac and pulmonary doses as well as treatment-related toxicities [186].

As radiotherapy becomes increasingly precise, the focus is shifting from sparing whole organs to selectively protecting smaller, functionally important substructures. This creates new opportunities to further reduce treatment-related toxicity, particularly for the heart, where growing evidence suggests that the risk of specific cardiac side effects depends on radiation exposure to distinct anatomical regions. However, the full potential of substructure-guided treatment planning can only be realized if the most clinically relevant cardiac endpoints and their underlying anatomical substrates are clearly identified. The findings of this review highlight that important knowledge gaps remain regarding which cardiac toxicities should be prioritized and which cardiac structures are most critical for their development. Addressing these questions is essential for translating technological advances into further improvements in patient outcomes. In this context, an ongoing prospective cohort study is investigating cardiac changes following thoracic radiotherapy and their dose–response relationships at the level of individual cardiac substructures [187].

Despite growing knowledge on radiation induced cardiac complications, significant methodological challenges continue to impede progress. Not all studies evaluated dose–response relationships for cardiac substructures, and there was substantial heterogeneity in the number and type of dosimetric parameters analysed. Restricting analyses to a limited set of parameters may obscure true dose–effect relationships and may have influenced the conclusions of this review. At the same time, many dosimetric variables are highly correlated [[74], [88], [112]], complicating interpretation. Additionally, the current evidence remains insufficient to support robust substructure-specific dose thresholds or clinically applicable planning recommendations for most cardiac structures. Although several candidate structures have been recognized, prospective studies using standardized imaging, contouring protocols and outcome definitions are needed to generate reproducible dose-response relationships and facilitate evidence-based dose optimisation. Current cardio-oncology guidelines may be used to facilitate routine cardiac screening and standardize endpoint definitions and imaging approaches [188]. Future studies should also adopt more robust analytical approaches, such as dimensionality reduction techniques or penalized regression models, to better disentangle the independent effects of individual dose parameters [189].

The impact of cardiac toxicity on survival is also complex. While multiple studies demonstrate associations between cardiac side effects and OS [[44], [131]], different toxicities have been pooled together, potentially obscuring the contribution of individual conditions. Analysing specific cardiac endpoints separately, or focusing on dose to critical substructures—as suggested in studies linking LAD or heart base dose to survival—may provide more clinically actionable insights.

Interventions in cardiac disease are more successful when performed in an early stage [[168], [188]]. However, cardiac side effects are often detected only after the onset of symptoms. Consequently, there is growing interest in identifying subclinical markers of cardiac injury, including serum biomarkers such as cardiac troponins and NT-proBNP, and imaging-based measures such as global longitudinal strain and coronary artery calcium progression. Cardiac troponin-T and NT-proBNP are sensitive early indicators of myocardial injury and stress [190], but they lack specificity, as elevated levels may also occur in non-cardiac conditions such as renal failure, sepsis and pulmonary embolism [[190], [191]]. In contrast, left ventricular GLS -which is a marker of myocardial wall deformation [192]- has shown potential for detecting subclinical systolic dysfunction [[152], [188]], and progression in coronary artery calcium (CAC) score on CT imaging have been associated with cardiac events after RT [193]. Despite these promising approaches, there is currently no consensus on how to incorporate these markers into clinical decision-making following RT [188].

Improved awareness and implementation of cardio-oncology guidelines, particularly those tailored to radiotherapy, may help bridge this gap. Current recommendations advocate regular cardiovascular risk assessment after cancer therapy, including clinical evaluation, biomarkers, and imaging in higher-risk patients [188]. However, awareness and adherence to these guidelines remain limited [[194], [195]], and radiotherapy-specific considerations are often underrepresented. Better integration of cardiology and oncology care, along with increased recognition of radiation-specific risks, is needed to translate existing knowledge into improved patient outcomes.

This study has several limitations. First, study screening, full-text assessment, and data extraction were performed by a single reviewer, which may increase the risk of selection and extraction errors. Nevertheless, the inclusion of 137 studies provides a broad overview of the available evidence on dose–response relationships for radiation-associated cardiac effects and their association with survival. Second, much of the available literature is retrospective and therefore susceptible to confounding, underreporting and selection bias. Third, heterogeneity in tumour types complicates interpretation as different tumour sites are associated with distinct cardiac dose distributions, prognosis, and competing risks, which may influence observed dose–response relationships. At the same time, inclusion of multiple thoracic malignancies enabled identification of common patterns of cardiac side effects across a broad range of clinical settings. Fourth, substantial variation exists in cardiac substructure contouring and dosimetric methodologies, potentially affecting reported dose metrics and limiting direct comparison across studies. These differences may influence reported dose metrics and limit direct comparison. Finally, differences in endpoint definitions and follow-up procedures further limit cross-study comparisons and interpretation of the available evidence.

## 5 Conclusion

Thoracic radiotherapy inevitably exposes portions of the heart and lungs to radiation. Accumulating evidence suggests that such exposure can affect cardiac structures and functions resulting in a broad spectrum of cardiac side effects. Furthermore, radiation dose to both cardiac and pulmonary tissues has been associated with reduced overall survival. As radiotherapy techniques continue to improve, minimizing radiation exposure to critical cardiopulmonary structures is becoming increasingly achievable. The findings of this review suggest that such dose reductions may not only decrease the risk of treatment-related toxicity but also contribute to improved long-term outcomes for patients undergoing thoracic radiotherapy.

## Funding

Peter van Luijk, Christina T. Muijs and Johannes A. Langendijk received funding from the Dutch Cancer Society under grant number 11349 (CLARIFY) and 14303 (MODELS).

## Declaration of generative AI and AI-assisted technologies

During the preparation of this work, Umut Fidan used ChatGPT to check grammar and improve text clarity. After using this tool, he reviewed and edited the content as needed and takes full responsibility for the content of the published article.

## Declaration of competing interest

The authors declare that they have no known competing financial interests or personal relationships that could have appeared to influence the work reported in this paper.
