# Supplementary material for: Dose-related cardiac side effects and its impact on survival following thoracic radiotherapy: A scoping review
Source: Clin Transl Radiat Oncol. 2026 Oct 6;62:101304. doi: 10.1016/j.ctro.2026.101304 (PMC13677307; doi:10.1016/j.ctro.2026.101304)
Supplement: Supplementary file 1 [file mmc1.docx]

**Supplementary Table 1: Search strategies by each database.**

Search strategy

PUBMED

("Thoracic Neoplasms"[Mesh] OR "Lung Neoplasms"[Mesh] OR "Esophageal Neoplasms"[Mesh] OR "Breast Neoplasms"[Mesh] OR "breast cancer"[tiab] OR "lung cancer"[tiab] OR "esophageal cancer"[tiab] OR "thoracic cancer*"[tiab] OR "hodgkin*"[tiab] OR "Lymphoma"[Mesh])

AND

(("Radiotherapy"[Mesh] OR "radiotherap*"[tiab] OR "radiation*"[tiab] OR "irradiat*"[tiab]) AND ("dose"[tiab] OR "dosage"[tiab] OR "dosi*"[tiab]))

AND

("Radiotherapy/adverse effects"[Majr] OR "Arrhythmias, Cardiac"[Mesh] OR arrhythm*[tiab] OR "Coronary Disease"[Mesh] OR (("cardiac"[tiab] OR "myocard*"[tiab] OR "pericard*"[tiab] OR "heart"[tiab]) AND (damage[tiab] OR injur*[tiab] OR toxic*[tiab] OR advers*[tiab] OR function*[tiab] OR dysfunct*[tiab] OR "infarct*"[tiab] OR "effusion"[tiab])))

Cochrane

([mh "Thoracic Neoplasms"] OR [mh "Lung Neoplasms"] OR [mh "Esophageal Neoplasms"] OR [mh "Breast Neoplasms"] OR "breast cancer":ti,ab OR "lung cancer":ti,ab OR "esophageal cancer":ti,ab OR ("thoracic" NEXT cancer*):ti,ab OR hodgkin*:ti,ab OR [mh Lymphoma])
AND
(([mh Radiotherapy] OR radiotherap*:ti,ab OR radiation*:ti,ab OR irradiat*:ti,ab) AND (dose:ti,ab OR dosage:ti,ab OR dosi*:ti,ab))
AND
([mh "Arrhythmias, Cardiac"] OR arrhythm*:ti,ab OR [mh "Coronary Disease"] OR ((cardiac:ti,ab OR myocard*:ti,ab OR pericard*:ti,ab OR heart:ti,ab) AND (damage:ti,ab OR injur*:ti,ab OR toxic*:ti,ab OR advers*:ti,ab OR function*:ti,ab OR dysfunct*:ti,ab OR infarct*:ti,ab OR effusion:ti,ab)))

Embase

('thoracic tumor'/exp OR 'lung tumor'/exp OR 'esophagus tumor'/exp OR 'breast tumor'/exp OR 'breast cancer':ti,ab,kw OR 'lung cancer':ti,ab,kw OR 'esophageal cancer':ti,ab,kw OR 'thoracic cancer*':ti,ab,kw OR 'hodgkin*':ti,ab,kw OR 'lymphoma'/exp) AND (('radiotherapy'/exp OR 'radiotherap*':ti,ab,kw OR 'radiation*':ti,ab,kw OR 'irradiat*':ti,ab,kw) AND ('dose':ti,ab,kw OR 'dosage':ti,ab,kw OR 'dosi*':ti,ab,kw)) AND ('radiotherapy'/exp/mj/dd_ae OR 'heart arrhythmia'/exp OR 'arrhythm*':ti,ab,kw OR 'coronary artery disease'/exp OR (('cardiac':ti,ab,kw OR 'myocard*':ti,ab,kw OR 'pericard*':ti,ab,kw OR 'heart':ti,ab,kw) AND ('damage':ti,ab,kw OR 'injur*':ti,ab,kw OR 'toxic*':ti,ab,kw OR 'advers*':ti,ab,kw OR 'function*':ti,ab,kw OR 'dysfunct*':ti,ab,kw OR 'infarct*':ti,ab,kw OR 'effusion':ti,ab,kw)))

NOT ('conference abstract'/it OR 'conference paper'/it OR 'conference review'/it)

Web of science core collection

("Thoracic Neoplasms" OR "Lung Neoplasms" OR "Esophageal Neoplasms" OR "Breast Neoplasms" OR "breast cancer" OR "lung cancer" OR "esophageal cancer" OR "thoracic cancer*" OR hodgkin* OR Lymphoma)
AND
((Radiotherapy OR radiotherap* OR radiation* OR irradiat*) AND (dose OR dosage OR dosi*))
AND
("Radiotherapy/adverse effects" OR "Arrhythmias, Cardiac" OR arrhythm* OR "Coronary Disease" OR ((cardiac OR myocard* OR pericard* OR heart) AND (damage OR injur* OR toxic* OR advers* OR function* OR dysfunct* OR infarct* OR effusion)))
